# Hip Disease in Children

**Published:** 1895-04

**Authors:** Samuel E. Milliken

**Affiliations:** Surgeon-in-Chief, New York Infirmary for Crippled Children; New York


For Texas Medical Journal.

### HIP DISEASE IN CHILDREN.

ABSTRACT OF A LECTURE BY DR. SAMUEL E. MILLIKEN, M. D.,
Surgeon-in-Chief, New York Infirmary for Crippled Children.

THE CLINICAL features of hip disease in children are varied, but the three cases spoken of, although representing different stages, were similar, in that none of them had had abscess.

*Hip Splint.*—The protection (extension) hip splint was employed. While it is impossible to produce complete immobilization with this splint, the movements of the hip joint are materially lessened. While this splint has the mechanism of the so-called extension apparatus, the greatest benefit obtained from it is that of protection. As yet we have no walking splint by which a uniform or constant amount of extension can be maintained.

The late Mr. H. O. Thomas, of England, has long ago demonstrated that with perfect immobilization and protection of the joint from concussion, very good results could be obtained. The greatest objection to his splint was that the patient had to walk on crutches. During the treatment of hip disease, careful measurements should be taken at least once a month. Where great muscular spasm exists, the patient should be kept recumbent, with the joint perfectly immobilized. Should that fail to relieve the reflex symptoms, Buck's extension should also be applied. By this means, the deformity can oftentimes be materially lessened, and thus be in better condition for wearing the protection splint. It is occasionally necessary to administer an anæsthetic for the purpose of correcting muscular spasm, after which the plaster-of-paris hip spica should be applied, and the joint kept quiet for ten days or two weeks before the hip splint is applied.

*Complications.*—Aside for the deformity which is produced by muscular spasm, we must not forget that ankylosis may be true or false, as is the case with other joints. If bony, a subtrochanteric osteotomy will be required; while if only fibrous or tendinous, the contracted structures can be divided.

*Abscess.*—Unless the abscess is so large that the mechanical treatment is interfered with, it should not be operated upon; however, should it develop rapidly, rather than allow an otherwise healthy structure to become involved, a free incision should be made, and the cavity drained, thus to avoid a series of disagreeable sinuses, which often follow the old method of allowing hip abscesses to evacuate voluntarily. Aspiration of these abscesses rarely proves effective, and often increases the suppurative process by the entrance of the external air.

*Excision.*—I am happy to say, the advocates of excision of the hip joint in children, are growing less each year, as experience has shown that conservative methods offer more favorable results, and the dangers of flail joint and retarded development are too well known to call for any further comment.

Attention to the general health during the treatment of tubercular joints, is of the greatest importance. Those engaged in special work are at times, no doubt, guilty of paying too little attention to the patient as a whole, and direct all their attention to the one particular lesion for which they were consulted. The digestion in these children is usually weak, and we should not expect large doses of cod-liver oil to be assimilated at first, but should be administered in small doses, and gradually increased.

The involvement of other joints and pulmonary tuberculosis, can be best prevented by keeping the general health in the best possible condition. Fresh air, and a long sojourn in the country during the summer months, works wonders with these anæmic subjects.

---

"The Excelsior Disinfector" is the name of a neat patented device for disinfecting and deodorizing. It is highly endorsed by leading physicians. The editor of this Journal is using one in his family with much satisfaction, and has recently given it a practical test in sick room. It dissipates or destroys all foul odor, and a pleasant aroma permeates the room afterwards. Price of the outfit is $2.75, with fluid enough for four months.

Mr. F. E. Huck, of Austin, is the agent for Travis county, to whom orders should be sent, or applications for further information.

